# Supplementary material for: Transcriptional patterns of sexual dimorphism and in host developmental programs in the model parasitic nematode Heligmosomoides bakeri
Source: Parasit Vectors. 2023 May 28;16:171. doi: 10.1186/s13071-023-05785-2 (PMC10225086; doi:10.1186/s13071-023-05785-2)
Supplement: Supplementary file 2 — Additional file 2. Supplementary figures and legends. [file 13071_2023_5785_MOESM2_ESM.pdf]

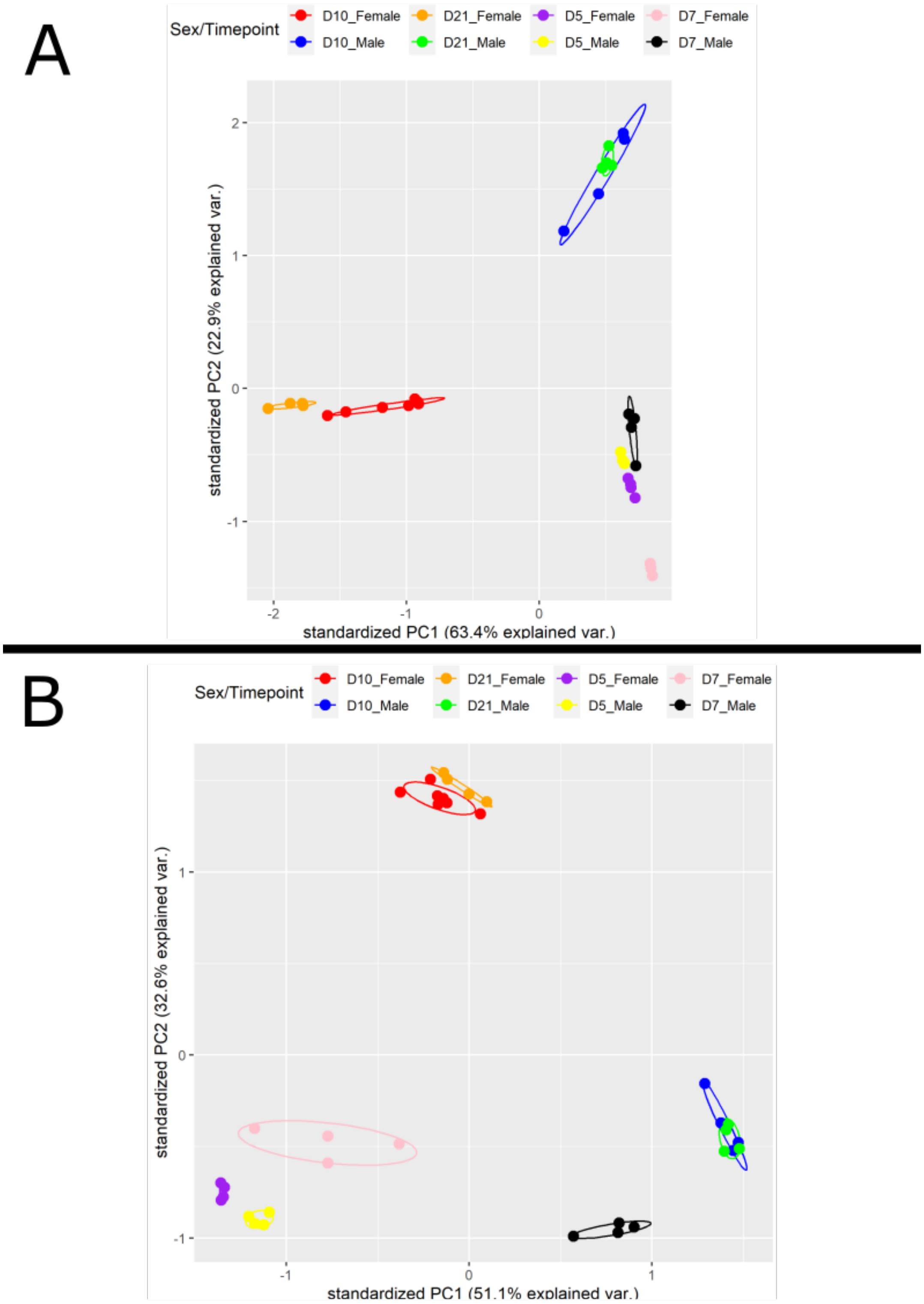

Fig S1. Principal component biplots of the RNA-seq datasets used in this study. A) Biplot of the first two components from the PCA of the raw counts. B) Biplot of the first two components from the PCA of the VST-transformed counts.

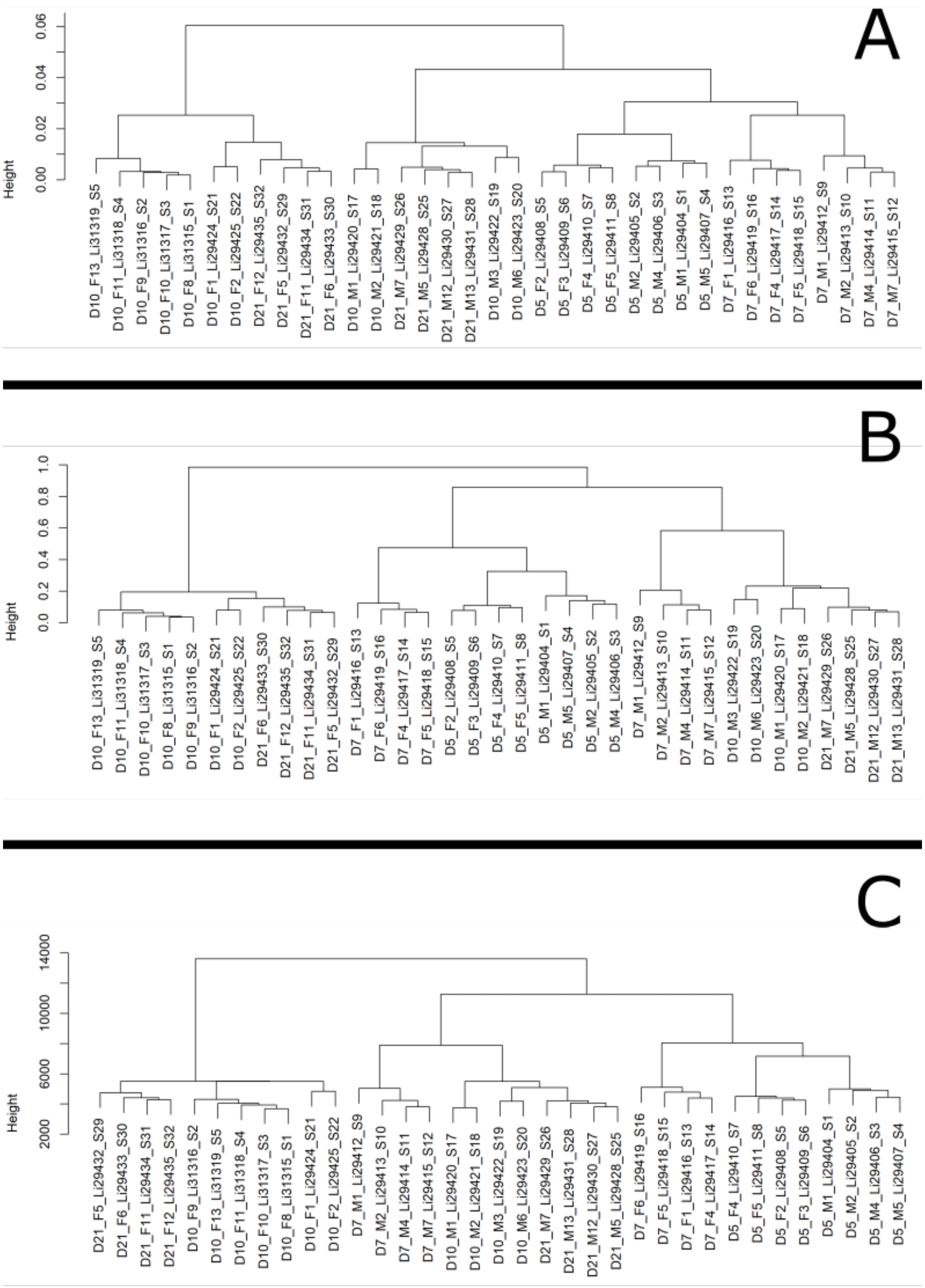

Fig S2. Hierarchical clustering dendrograms of the datasets used in this study. A) HC using Euclidean distance and average linkage. B) HC using Manhattan distance and average linkage. C) HC using Canberra distance and average linkage.

Unsigned Co-expression Network Modules

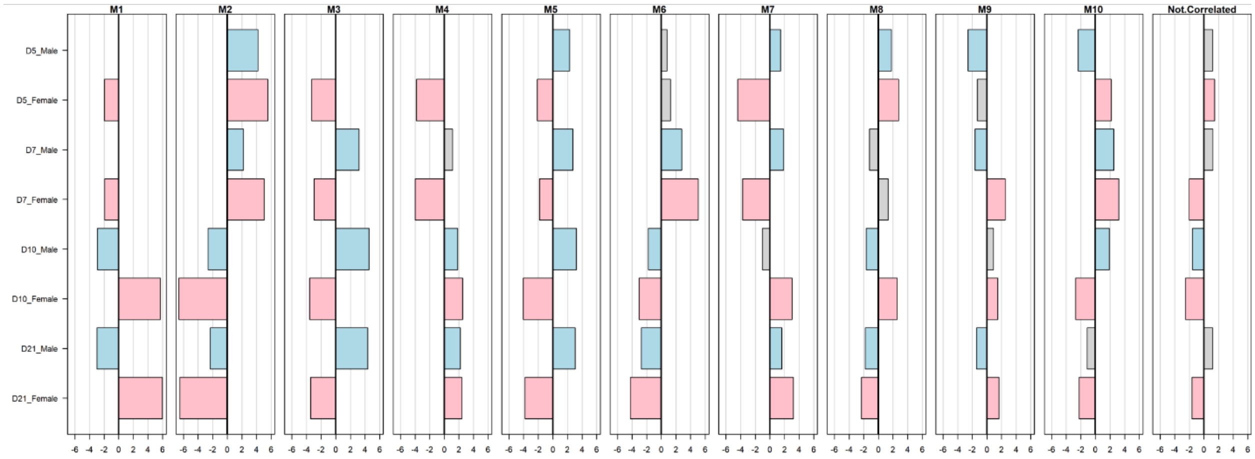

Signed Co-expression Network Modules

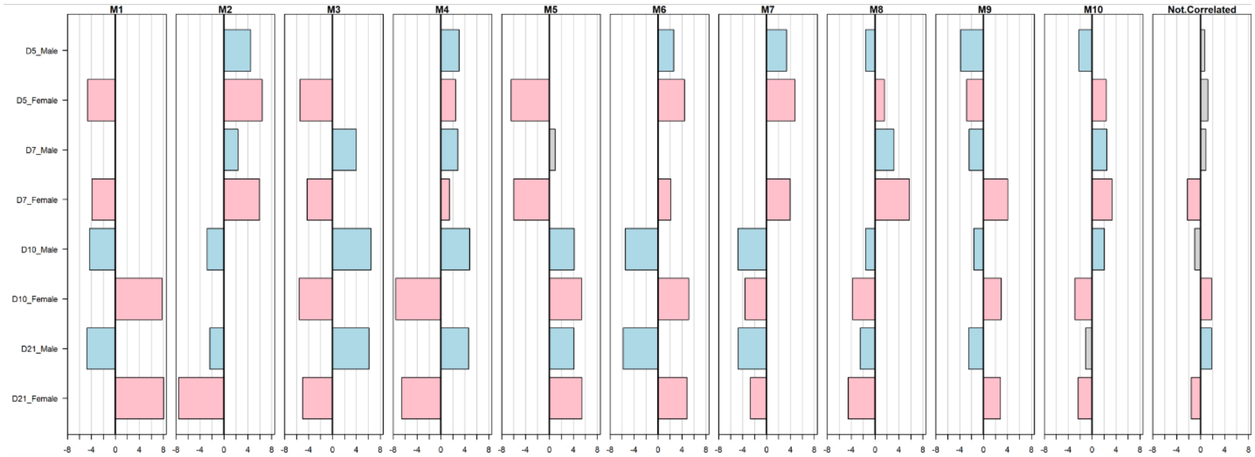

Fig S3. Transcript co-expression networks. Normalized enrichment scores, reflecting the expression values of the genes in the module, are plotted for each module returned by the network. Unsigned networks allow strong negative correlations to be connected within a module, whereas signed networks do not. Both are shown.

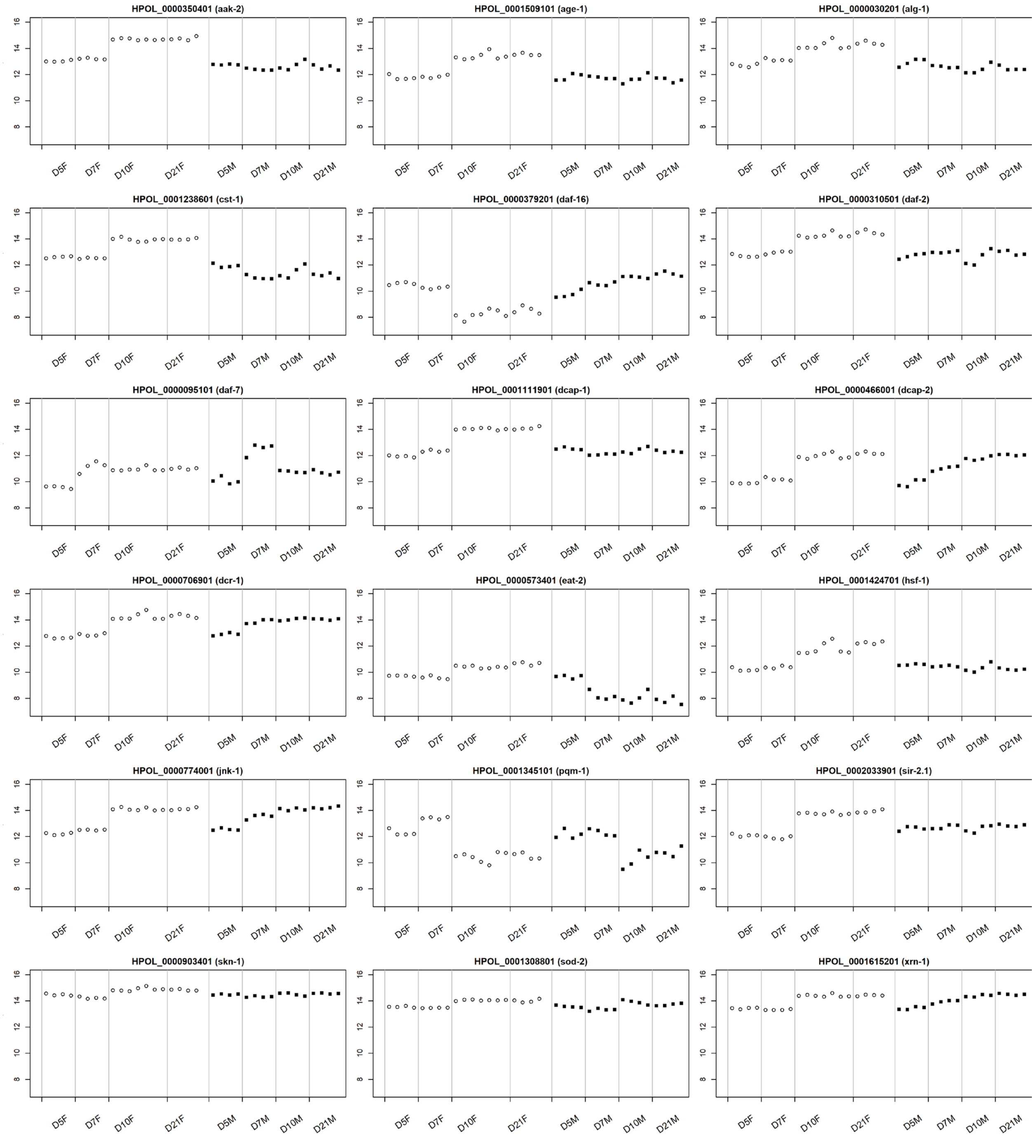

Fig S4. Expression of orthologs of genes in *C. elegans* that have been implicated in aging. Scatterplots of the VST-transformed read counts (which estimate expression level of the transcript) for all samples grouped by their age/sex combination. The transcript in *H. bakeri* and ortholog in *C. elegans* are shown above the plot.

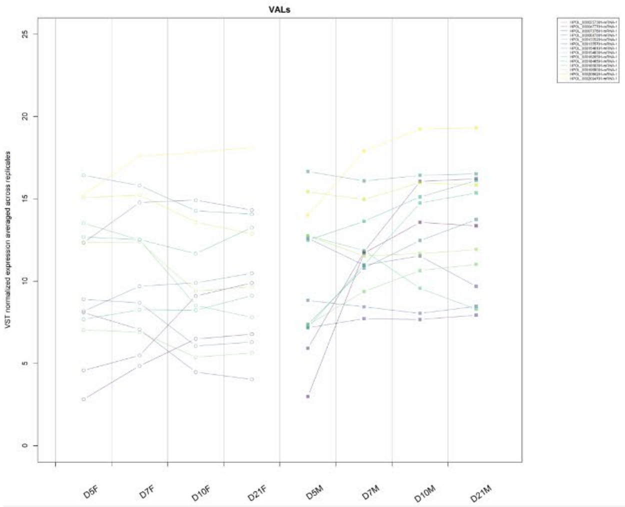
VALs
VST normalized expression averaged across replicates
0
5
10
15
20
25
D5F
D7F
D10F
D21F
D5M
D7M
D10M
D21M

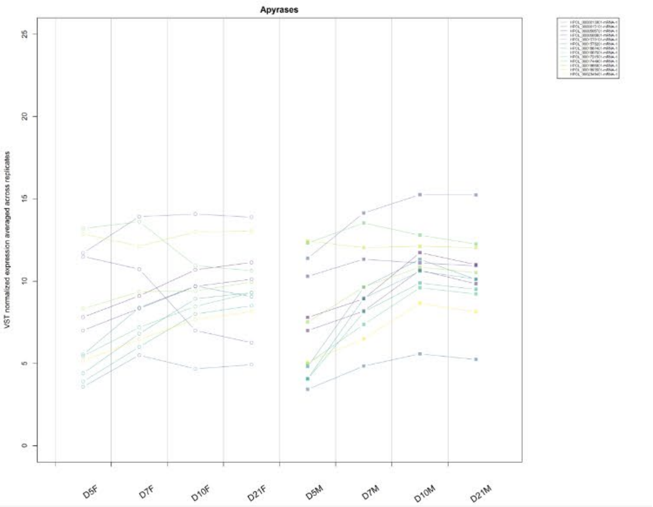
Apyrases
VST normalized expression averaged across replicates
0
5
10
15
20
25
D5F
D7F
D10F
D21F
D5M
D7M
D10M
D21M

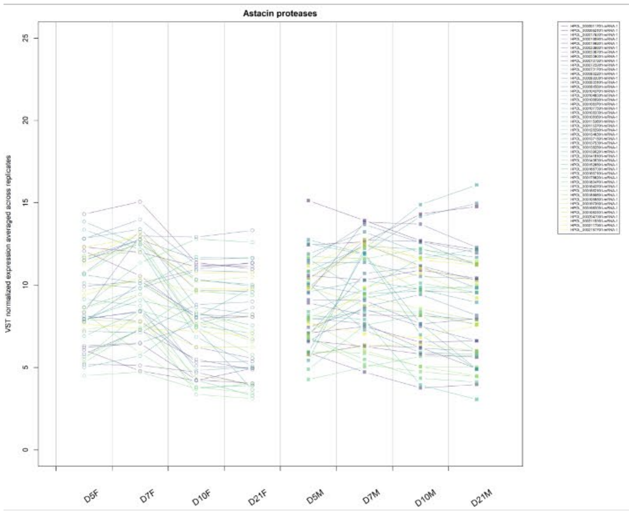
Astacin proteases
VST normalized expression averaged across replicates
0
5
10
15
20
25
D5F
D7F
D10F
D21F
D5M
D7M
D10M
D21M

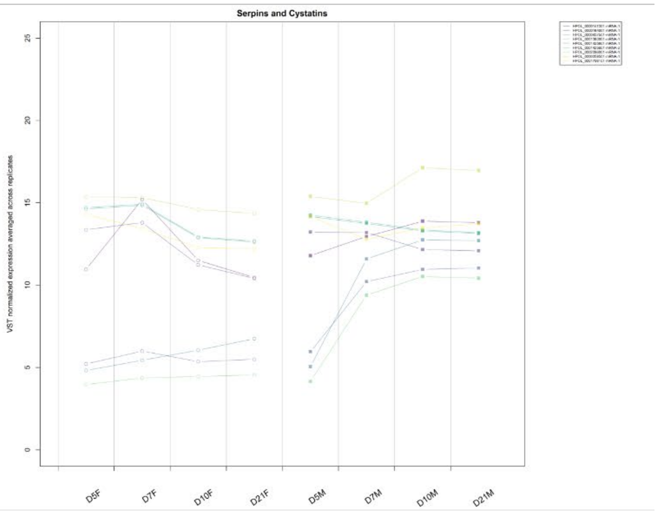
Serpins and Cystatins
VST normalized expression averaged across replicates
0
5
10
15
20
25
D5F
D7F
D10F
D21F
D5M
D7M
D10M
D21M

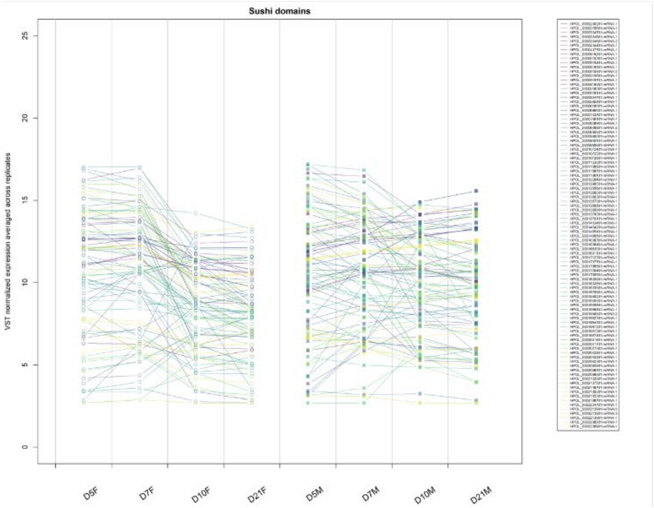
Sushi domains
VST normalized expression averaged across replicates
0
5
10
15
20
25
D5F
D7F
D10F
D21F
D5M
D7M
D10M
D21M

Fig S5. Expression of immunomodulatory genes. The arithmetic mean for each age/sex group of the VST-transformed read counts (which estimate expression level of the transcript) are plotted for every transcript matching the description above each plot. Average expression values for female samples are on the left and transcripts are plotted with an open circle connected by lines. Average expression values for male samples are on the right and transcripts are plotted with closed squares connected by lines. Symbols and lines are colour coded to indicate the transcript being plotted with each being labelled in the legend to the right of the plot.
