# Supplementary material for: Transcriptional patterns of sexual dimorphism and in host developmental programs in the model parasitic nematode Heligmosomoides bakeri
Source: Parasit Vectors. 2023 May 28;16:171. doi: 10.1186/s13071-023-05785-2 (PMC10225086; doi:10.1186/s13071-023-05785-2)
Supplement: Supplementary file 6 — Additional file 6. Compressed file of all the images used for the bead-feeding assay. [file 13071_2023_5785_MOESM6_ESM.pdf]

Additional File 6: Compressed file of all the images used for the bead-feeding assay

Given the large size of the dataset it is available on Data Dryad

doi:10.5061/dryad.ttdz08m2x

doi.org/10.5061/dryad.ttdz08m2x
